# Inhaled nitric oxide clinical confusions: population types, duration, and responsiveness

**DOI:** 10.1186/s13054-024-05181-x

**Published:** 2024-11-25

**Authors:** Kai Liu, Shi-Min Zhang, Jing-chao Luo, Min-jie Ju

**Affiliations:** 1 Department of Critical Care Medicine, Zhongshan Hospital, Fudan University, No. 180 Fenglin Road, Xuhui District, Shanghai, 200032 China; 2 Department of Critical Care Medicine, Sichuan Academy of Medical Sciences and Sichuan Provincial People’s Hospital, University of Electronic Science and Technology of China, Chengdu, 610072 China

To the Editor,

Inhaled nitric oxide (iNO) is widely used to treat hypoxic patients, especially those with acute respiratory distress syndrome (ARDS) [1]. We read with great interest the study by Isha et al. investigating the therapeutic efficacy of low-dose iNO in patients with COVID-19 [2]. Although this study provides valuable insights into this therapeutic approach, several considerations merit detailed discussion.

The factors that influence the effectiveness of iNO include patient population heterogeneity, and previous multicenter studies have reported response rates ranging from 20 to 60% [3]. Similar differences are observed in COVID-19 patients, possibly due to biological factors, such as such as the suppressed endogenous nitric oxide, across various populations [4]. In Isha’s study, the authors used multiple levels of categorization. This included initial grouping based on breathing status (spontaneous vs. intubated), followed by subdivisions considering factors such as progression to intubation, iNO usage, the timing of iNO initiation (pre- or post-intubation), early intubation (< 5 days), and iNO use for more than 48 h before intubation. However, the absence of patient-self stratification, along with a complex system that focused on the type of respiratory support, posed significant challenges to interpreting the results. Additionally, this extensive stratification likely resulted in small subgroup sizes, which may have compromised statistical power. Future trials should focus on well-defined patient subgroups, rather than on broad, heterogeneous populations.

The effect of iNO on oxygenation varies over time, making the duration of iNO treatment an important factor. Long-term iNO inhalation can lead to time-dependent changes in drug effects, with patients gradually developing stronger responses to lower doses of NO [5]. In Isha’s study, the duration of iNO administration was not specified, especially for those in the spontaneously breathing group who received iNO before intubation. It is unclear whether these patients continued iNO treatment after intubation, which could affect the results.

The response criteria for iNO in hypoxic patients are crucial. The widely accepted criterion for iNO effectiveness is a 20% increase in PaO_2_, PaO_2_/FiO_2_, or the oxygenation index (within 30 min to 1 h after iNO initiation), which serves as a reliable indicator for evaluating the immediate treatment effect [5, 6]. In contrast, the authors defined a response as an increase in PaO2/FiO2 or a decrease in FiO2 within 48 h. This 48-h extended assessment and the uncertainty in the range of oxygenation enhancement may have influenced the estimation of immediate physiological responses to iNO, potentially affecting the interpretation of outcomes, particularly in the absence of iNO duration data.

Finally, we appreciate the authors' efforts in conducting this research. While iNO showed a positive impact, and future studies should focus on well-defined patient populations, clearly documented iNO administration durations, and precise response criteria to more accurately evaluate the therapeutic benefits of iNO.

Sincerely,

## Data Availability

No datasets were generated or analysed during the current study.
